# Brain Imaging in Patients with Non-Small Cell Lung Cancer—A Systematic Review

**DOI:** 10.3390/jcm14030708

**Published:** 2025-01-22

**Authors:** Nora Mayer, Laura Boschetti, Marco Scarci, Ugo Cioffi, Matilde De Simone, Marlène Schnider, Peter Kestenholz, Fabrizio Minervini

**Affiliations:** 1 Division of Thoracic Surgery, Cantonal Hospital Lucerne, 6000 Lucerne, Switzerland; nora.mayer@luks.ch (N.M.); marlene.schnider@gmail.com (M.S.); peter.kestenholz@luks.ch (P.K.);; 2 Department of Medical Oncology, Cantonal Hospital Lucerne, 6210 Sursee, Switzerland; 3 Division of Thoracic Surgery, Imperial College NHS Healthcare Trust and National Heart and Lung Institute, London W2 1NY, UK; marco.scarci@nhs.net; 4 Department of Surgery, University of Milan, 20122 Milan, Italy

**Keywords:** non-small cell lung cancer (NSCLC), preoperative staging, brain metastases, brain imaging, MRI

## Abstract

**Background**: Lung cancer frequently metastasizes to the brain, liver, and adrenal glands with a significant negative prognostic impact on overall survival and quality of life (QoL). To optimize treatment and prognosis, adequate staging with the detection of distant metastases is crucial. The incidence of brain metastases in potentially resectable early-stage non-small cell lung cancer (NSCLC) is as low as 3%; hence, the need for preoperative brain imaging has been a constant matter of debate, especially in stage II. In stages III and IV NSCLC, neuroimaging is an essential part of staging. **Methods**: A systematic literature search was performed. Publications from 1999 to 2024, focusing on preoperative brain imaging (BI) in the staging of stages I–IV NSCLC, were included. Data extraction included study population characteristics, the modality of BI, the incidence of brain metastases (BMs), and the main outcomes of the studies. The final included studies were selected according to the PRISMA criteria. In the second step, guidelines on BI in NSCLC staging of major importance were identified and compared. **Results**: A total of 530 articles were identified, of which 25 articles were selected. Four prospective studies and 21 retrospective investigations were included. Most of the investigations focused on BI in the early stages. The main imaging modality for BI was magnetic resonance imaging (MRI), followed by computed tomography (CT). Besides the identified 25 studies, the most important internationally applied guidelines on brain imaging in the staging of NSCLC were reviewed. While some guidelines agree on preoperative BI in NSCLC stage III (Union for International Cancer Control—UICC eighth edition) patients, other guidelines recommend earlier BI starting from clinical stage II. All mentioned guidelines homogenously recommend BI in patients with symptoms suggestive of brain pathologies. **Conclusions**: BI in NSCLC staging is recommended in neurologically symptomatic patients suggestive of brain metastases as well as NSCLC patients with stage III disease. Neuroimaging in stage IA patients, as well as in pure GGO (Ground-Glass Opacity) lesions, was considered unnecessary. The predominantly applied imaging modality was ce-MRI (contrast-enhanced magnetic resonance imaging). Inconsistency exists concerning BI in stage II. The identification of prognostic factors for developing BM in patients with early-stage NSCLC could help to clarify which subgroup might benefit from preoperative BI.

## 1. Introduction

Lung cancer remains the most common cause of cancer-related deaths worldwide [1,2,3]. Among lung cancer, non-small cell lung cancer (NSCLC) is the leading cause of brain metastases (BMs) (40–50%) [4,5]. BMs are present in 10–20% at initial diagnosis, and up to 40% of the patients develop BMs during the course of their disease [6,7,8,9]. BMs are known to significantly increase morbidity and mortality and reduce patients’ quality of life (QoL) [4,10]. The incidence of BMs was found to increase in accordance with the overall stage, a higher nodal stage and the presence of N3-disease, the existence of distant metastatic disease (M1b), and the histology of adenocarcinoma [11,12].

Most BMs are located in the cerebral parenchyma and are best diagnosed with magnetic resonance imaging (MRI) [13]. Gadolinium-enhanced MRI (ce-MRI) is widely used for screening purposes, providing a higher sensitivity and specificity than contrast-enhanced computed tomography (ce-CT) in detecting BMs [13]. Nevertheless, in real life, ce-CT still plays a role in brain imaging (BI) due to contraindications to MRI like claustrophobia, magnetic implantable medical devices, or restricted MRI availability [14].

Only 25% of individuals diagnosed with BMs in MRI were clinically symptomatic at diagnosis, and clinical evaluation including a thorough history and physical examination remains the best predictor of metastatic brain disease [15].

QoL and overall survival (OS) are negatively affected by the presence of BMs, with an OS range reported from six to nine months depending on diverse treatment strategies and stage at diagnosis [4,10,16]. Earlier on, the median survival from diagnosis of BMs to death was described as approximately one to two months without treatment [17]. QoL and OS could be significantly improved with the treatment of BMs involving medical treatment (steroids), surgery, and radiotherapy of the neurocranium [18,19]. The central role of BI for locally advanced or symptomatic patients is well-defined. Nevertheless, guidelines for neuroimaging, especially in early NSCLC stages in the preoperative setting, are still a controversial topic.

While the currently existing international guidelines, i.e., the ESMO (European Society for Medical Oncology), NCCN (National Comprehensive Cancer Network), NICE (National Institute for Health and Care Excellence), BTS (British Thoracic Society), and ACCP (American College of Chest Physicians) guidelines, concordantly recommend screening for stage III NSCLC patients, the recommendations vary for the indication of BI in early stages [20,21,22,23,24,25].

Early diagnosis and control of BMs in NSCLC are important for adequate staging and therapy, especially given the constantly increasing diversity of locally and systemically applicable therapeutic options [26]. However, as summarized above, several guidelines present inconsistent recommendations regarding routine preoperative BI, especially in clinical stages I and II NSCLC. For early stages, consensus only exists for BI not being necessary for the staging of pure ground glass nodular lung adenocarcinoma [27].

In our systematic review, we aim to summarize the available evidence on preoperative neuroimaging in the staging of NSCLC. First, we provide an overview of all the published and relevant literature and studies investigating preoperative BI in NSCLC stages I-IV patients. Second, the existing guidelines for BI in the staging of NSCLC are compared and evaluated based on the identified studies. In the last step, we finally aim to provide an additional recommendation to the existing guidelines on indications for neuroimaging in NSCLC staging.

## 2. Materials and Methods

### 2.1. Literature Research Strategy

Systematic literature research was conducted between 10th of March and 15th of March 2024, according to the updated PRISMA (“Preferred Reporting Items for Systematic Reviews and Meta-Analyses”) 2020 guidelines for reporting systematic reviews, in the PubMed, MEDLINE, Embase, CENTRAL, and CINAHL databases to identify relevant publications on BI in NSCLC, including publications from 1999 to February 2024.

Search terms including Boolean operators were used, as listed below:

(Imaging OR brain imaging OR MRI OR magnetic resonance imaging OR CT OR computed tomography OR PET-CT) AND (brain metastasis OR brain metastases) AND staging AND (NSCLC OR non-small cell lung cancer). Further articles were identified through a manual search of the reference lists of articles identified through the original search.

Data were summarized using descriptive statistics. Data were analyzed and presented as a proportion of the total. Categorical variables were reported as percentages.

### 2.2. Inclusion and Exclusion Criteria

Studies focusing on BI in NSCLC staging throughout all stages according to the 6th, 7th, or 8th TNM edition (UICC), published between 1999 and February 2024, were included. Manuscripts published earlier than 1999 were excluded, unless they were of paramount significance for the included staging guidelines (NICE, BTS, NCCN, ACCP, and ESMO).

Manuscripts focusing on BI (a) after surgery with curative intent for NSCLC, (b) BI in long-term follow-up after surgery, and (c) studies focusing on economic aspects of BI in NSCLC staging were not included in the main review process. Further exclusion criteria were manuscripts in the form of commentaries, case reports, editorials, and surveys. Publications in languages other than English and full-text unavailability were also excluded. In order to homogenize the evaluated patient populations, certain manuscripts of importance for this systematic review were included, but only the patient cohort with BI performed during preoperative staging was finally included in the analysis, while patients without neuroimaging or BI in follow-up were not included [28].

### 2.3. Data Extraction and Critical Appraisal of Evidence

All references returned from the above searches were exported into a ZOTERO library.

Initially, records were screened by title and abstract, and duplicate studies were identified and removed. In the second stage of the screening, we performed a full-text review of all eligible studies from the title and abstract screening. Both stages were performed by three authors (NM, LB, FM). Information was collected on study design, country of study and patient recruitment, number of included patients, gender, histologic type of lung cancer, the incidence of brain metastases (BMs), imaging frequency and modality, the share of upstaged patients due to BMs, and the study conclusions.

## 3. Results

### 3.1. Study Selection

**Figure 1:**
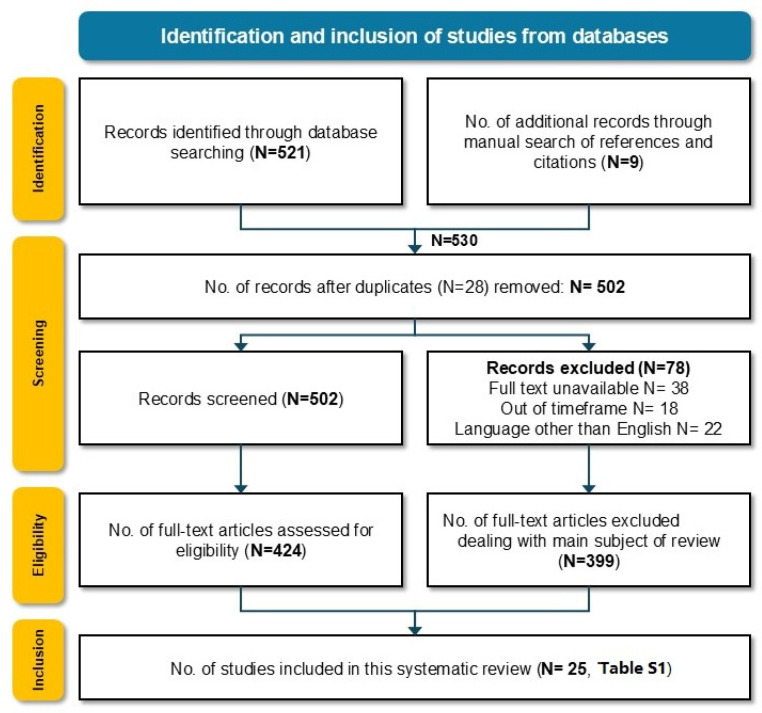
Identification and selection of studies according to The Preferred Reporting Items for Systematic Reviews and Meta-Analyses (PRISMA) statement criteria for the systematic review process. Abbreviations: No. = number of.

The literature research identified 530 articles, of which 502 were screened following the removal of duplicates. A total of 424 full-text reviews were performed in accordance with our inclusion and exclusion criteria. Following the critical appraisal, a total of 25 articles were included in this systematic review. Figure 1 illustrates the study selection process according to the Preferred Reporting Items for Systematic Reviews and Meta-Analyses (PRISMA) statement guidelines [29]. The design of the identified studies, year of recruitment and publication, NSCLC stages, inclusion and exclusion criteria, modality and frequency of BI, the incidence of BMs, and histologic tumor results, as well as the main outcomes, upstaging due to BM, and recommendations given by the authors, are summarized in Table S1.

### 3.2. Risk of Bias Assessment

**Figure 2:**
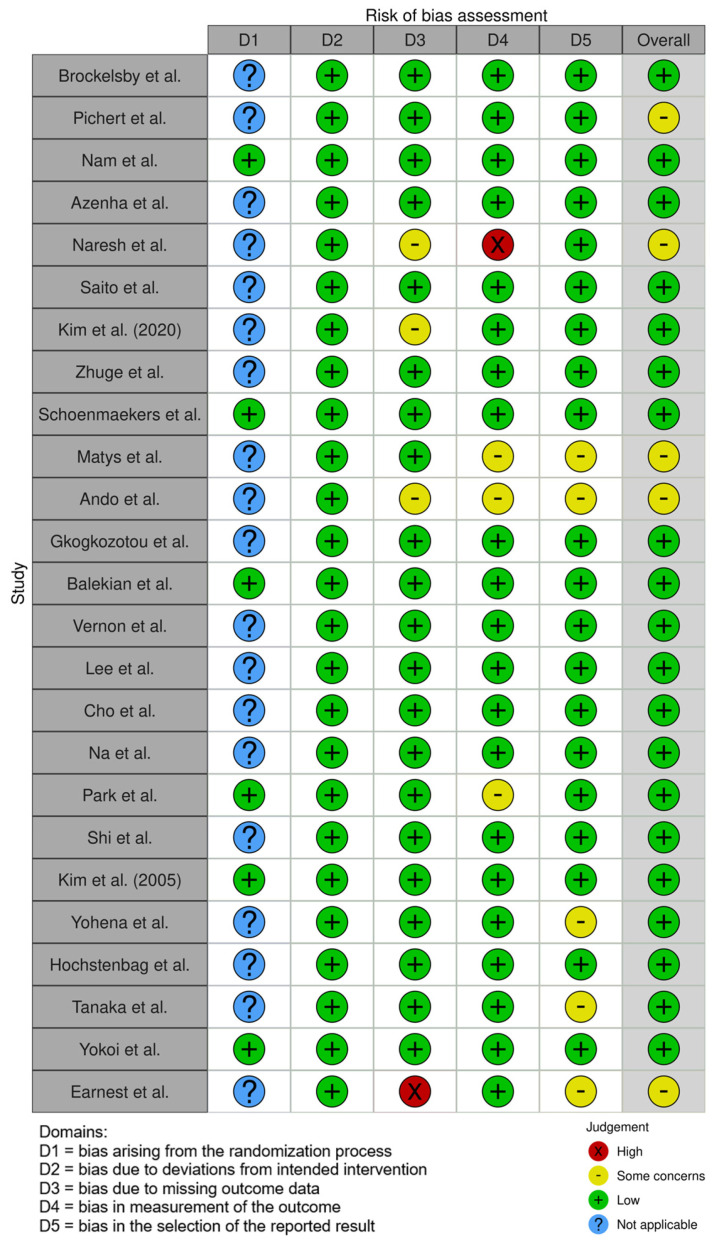
Risk of bias assessment performed using the robvis generic tool. Risk of bias was defined as high, some concerns, low, or not applicable. Domains D1 = bias arising from the randomization process, D2 = bias due to deviation from the intended intervention, D3 = bias due to missing outcome data, D4 = bias in the measurement of the outcome, and D5 = bias in the selection of the reported results were assessed [11,12,15,27,28,31,32,33,34,35,36,37,38,39,40,41,42,43,44,45,46,47,48,49,50].

A summary of the risk of bias assessment for the 25 included studies using the GENERIC robvis tool is shown in Figure 2 [30]. Only six studies were of a prospective nature and thus could be evaluated for randomization bias. None of the studies were rated to be at a critical risk of bias; however, five studies were rated with some concerns of being at a risk of bias. The overall risk of bias was low for the majority of the included studies. Across all studies, bias due to deviation from the intended intervention was the criterion least susceptible to bias (Figure 2).

### 3.3. Summary Findings of the Included Studies

A total of 25 publications were included in this systematic review. Four prospective studies were included in addition to 21 retrospective investigations. The reported stages according to the three different TNM staging systems (sixth to eighth) were reported for transparency reasons and adequate comparison. The reported modalities for cerebral imaging were ce-MRI and ce-CT. Most of the studies investigated patients diagnosed with stage I, followed by stages II, IIII, and IV. In the systematically reviewed studies, preoperatively diagnosed BMs were reported to occur with overall incidences of 0% to 20.1%, while increasing from 0 to 3.8% in stage I, 2.1–8.5% in stage II, 1–9% to 6.7% in stage III, and 11.1% to 20.1% in stage IV (Table S1). The main conclusions drawn by the authors of the respective studies are summarized in the last column. The authors consistently agreed on the unnecessity of BI in stage IA NSCLC, unless in EGFR mutated patients. BI in stages III–IV NSCLC was claimed to be essential, as reflected in the existing guidelines.

### 3.4. Imaging Modalities for the Detection of Brain Metastases

#### 3.4.1. Magnetic Resonance Imaging (MRI)

**Figure 3:**
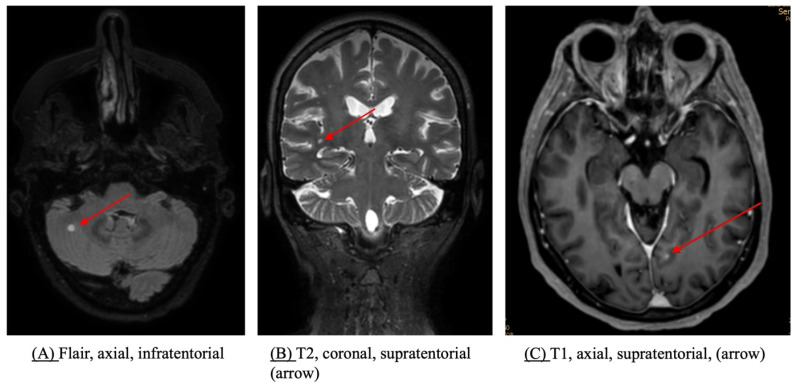
MRI in the detection of BMs. Red arrows indicate the identified BM in different MRI modalities and locations with an (**A**) infratentorial metastasis in flair, (**B**) a supratentorial metastasis in the T2-sequence, and (**C**) a paramedian metastasis in the T1-sequence. Abbreviations: BM = brain metastases, MRI = magnetic resonance imaging.

In the included studies, contrast-enhanced ce-MRI (Figure 3) was the method of choice to detect cerebral metastases [44,50]. While eight investigations performed neuro-staging with both CT and MRI, only one study used ce-CT as their imaging modality of choice [38].

Ce-MRI brain showed a very high sensitivity and specificity for the detection of BMs with 97.7% and 100%, respectively [46]. The preoperative ce-MRI detection rate of BMs was higher than with ce-CT [37]. Ce-MRI was specifically superior to ce-CT head for the detection of small (<1 cm) BMs, posterior fossa lesions, and multiple lesions [15,51,52,53]. The published guidelines uniformly recommend ce-MRI as the first choice for brain imaging, with ce-CT being an alternative in cases with MRI unavailability and for prevention of delayed treatment (Table 1).

#### 3.4.2. Contrast-Enhanced Computed Tomography (ce-CT)

**Figure 4:**
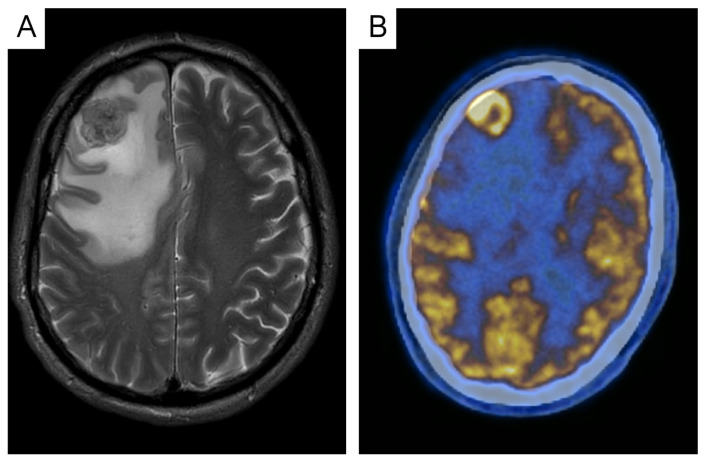
ce-CT vs. PET-CT in the detection of brain metastases (BMs). A large right frontotemporal BM with surrounding edema was clearly diagnosed in the ce-CT head (**A**), while PET-CT was suggestive of an FDG-avid lesion in this area (**B**). Abbreviations: ce-CT = contrast-enhanced computed tomography, PET-CT = positron emission computed tomography.

Only one of the identified studies mentioned ce-CT (Figure 4) as the first choice for BI. In contrast, 32% of the authors reported ce-CT to be equally applicable to ce-MRI in brain imaging, and 68% of the studies performed neuroimaging using MRI.

Prior to the introduction and more widespread use of ce-MRI for investigating the brain, ce-CT head was applied in staging for BM [15,34]. False positive diagnoses of BMs with ce-CT head were reported in up to 11% of the screened patients, and ce-CT was shown to be less sensitive than ce-MRI in detecting small metastases [51,55].

#### 3.4.3. Positron-Emission Computed Tomography (PET-CT)

The sensitivity and specificity to detect distant metastases in NSCLC were 93% and 96%, respectively; however, the sensitivity for BMs was only 60% [21,55]. The problem with BMs in PET-CT arises firstly due to the small size of most BMs and the background brain F-fluoro-2-deoxy-D-glucose (FDG) uptake, which can cover the presence of BMs [20]. However, De Wever et al. argued, in their small series including 87 patients, that CT was superfluous for the detection of BMs when PET-CT was available [56]. Moreover, PET-CT was found to decrease unnecessary surgery through more accurate staging while still being somewhat cost-effective [57]. In the systematically reviewed studies, Gkogkozotou et al. reported PET-CT and MRI brain in combination to be sufficient for correct staging, and Vernon et al. even claimed neuro-MRI to be superfluous in the presence of ce-CT chest and PET-CT [40,42]. Cho et al. showed that PET-CT in GGO was of no diagnostic value [27].

#### 3.4.4. MRI-PET

MRI-PET is a new hybrid technique that reduces radiation doses by about 31%. Lee et al. demonstrated that MRI-PET in combination with ce-CT was comparable to PET-CT in the preoperative staging of NSCLC [58]. However, MRI-PET was not found to be superior to PET-CT plus brain MRI in preoperative staging in NSCLC patients [59,60].

Deuschl et al. evaluated 18F-FDG PET/MRI in comparison to MRI alone and found that 18F-FDG MRI-PET does not lead to an improvement in diagnostic accuracy in the CNS staging of NSCLC patients [61]. None of the identified and included studies implemented MRI-PET in neuroimaging in the context of NSCLC staging.

### 3.5. Existing Guidelines on Preoperative Brain Imaging in NSCLC Patients

#### 3.5.1. American College of Chest Physicians (ACCP) 2013

According to the ACCP guidelines, routine imaging with MRI or ce-CT (when MRI is not available) is recommended in stages III and IV [ 21] Moreover, neurologically symptomatic patients should undergo brain screening. Furthermore, the ACCP mentions that biannual follow-up MRI brain may detect early BMs, thereby providing opportunities for radio-surgery [62].

#### 3.5.2. British Thoracic Society (BTS) 2010

The British Thoracic Society (BTS) guidelines from 2010 recommend an MRI or CT scanning of the head in stage III and generally in all NSCLC stages when patients are eligible for radical therapy with curative intent [21,25]. Furthermore, patients with features suggestive of intracranial pathology should be evaluated by an initial CT scan of the head followed by MRI if CT is normal, or MRI should be performed as an initial test in symptomatic patients.

#### 3.5.3. European Society for Medical Oncology (ESMO) 2017

The ESMO guidelines recommend BI with MRI or optional ce-CT in stage III NSCLC, according to the eighth edition of the TNM staging system [63]. In patients with stages I and II NSCLC, a preoperative BI is considered useful but not mandatory; therefore, a clear recommendation is lacking [22].

#### 3.5.4. National Comprehensive Cancer Network (NCCN) 2018

The NCCN guidelines do not advise BI in stage IA. In stage IB, it is considered optional, and it is recommended from stage II to higher stages, specifically with MRI [24].

#### 3.5.5. National Institute for Health and Care Excellence (NICE) 2011

The NICE and BTS guidelines recommend an MRI or ce-CT screening from stage III upwards and generally in all stages when patients are eligible for therapy with curative intent [21,25].

#### 3.5.6. Summary and Comparison of Currently Existing Guidelines

There is consensus across ESMO, ACCP, and BTS guidelines on preoperative BI in NSCLC stage III (UICC eighth edition) patients [20,21,22]. However, the NCCN and NICE guidelines recommend earlier neuroimaging, also screening patients with suspected lower clinical stage II [24,25]. While NCCN and NICE guidelines advise against BI in stage I in equal measure, the ESMO and BTS guidelines state vaguely that BI might be conducted in patients of any stage if considered for curative surgery. All guidelines further recommend BI in patients with features suggestive of brain pathologies.

While the ACCP guidelines mention biannual MRI brain following NSCLC resection with curative intent, there is no mention of BI in follow-up in NSCLC in any of the other guidelines (Table 1).

## 4. Discussion

Adequate staging in NSCLC patients is crucial for choosing the most appropriate treatment option within a reasonable time frame to avoid delay of diagnosis and commencement of treatment [25]. Still, preoperative neuroimaging remains a highly controversial topic, especially in early stages I and II, while there is agreement on stages III and IV with mandatory BI recommendations across all the existing guidelines.

In the aforementioned guidelines (NCCN, BTS, ACCP, ESMO, NICE), consensus exists on the fact that ce-MRI brain is the most sensitive imaging modality in detecting BMs, followed by ce-CT head in cases of MRI unavailability or contraindication (Table 1).

However, an EORTC (European Organization for Research and Treatment of Cancer) survey conducted in 2018 revealed that European BM management is often not performed according to European guidelines [64]. This issue reoccurred in a Dutch study by Brockelsby et al., with 32% of stage III patients not receiving pre-treatment BI [31]. This fact stresses the need for uniform guidelines that are appropriately applied.

The most controversially discussed topic that remains is whether stage II should be routinely screened for BMs preoperatively, as recommended by the NCCN and NICE guidelines, and whether this practice is still considered cost-effective.

Supporting the recommendation of NCCN and NICE to screen stage II, Pichert et al. just recently showed, in 2022, that the risk for BMs was about 6% in both clinical stage II and III in about 7000 newly diagnosed NSCLC patients. There was even a little drop in incidence down to 5% in stage III as compared to 6.4% in stage IIA [12]. Of note, the study population totaled less than approximately 150,000 patients, which, to our knowledge is the second largest number of data sets evaluated on this topic and certainly the most thorough investigation of the latter [12,65]. Matys et al. retrospectively showed that 2.6% of their evaluated stage IIIA patients vs. 3% (IIA) and 4.3% (IIB) had BMs at initial staging (diagnosed with ce-CT), certainly again raising the need for questioning the guidelines that only recommend BI in stage III [38]. Brockelsby et al. most recently backed up the above findings by recommending routine screening in stage II in their multicenter study conducted in the UK. In their study, 6% of NSCLC stage III and 6.2% of stage II patients were diagnosed with BMs [31]. Lee et al. also reported similar incidences of BMs in stage IIA of 2.4%, IIB of 3.8%, and IIIA of 1.9%. Stage IIIB patients showed BMs in 5.9% of the squamous cell lung cancer cases [58].

However, there is conflicting evidence about the incidence of BMs in stage II. In contrast to the abovementioned studies, highlighting the fact that BI in the clinical staging of NSCLC should be performed in patients in stage II, the incidence of BMs in stage II was also reported to be rather comparable to the incidences in NSCLC stage I. According to Tanaka et al., only around 2% of patients had BMs in stage T2N0 (IIA) [49]. Saito et al. recently found that only 3% of patients in stage II (IIA, T2N0) had BMs [33]. Hochstenbag et al. finally reported that the incidence of BMs in patients with large cell carcinoma or adenocarcinoma was 3% in stages I and II but about 21% in stage IIIA [48].

Asymptomatic BMs in stage I NSCLC have been reported in up to 3.8% of patients [20]. However, in a retrospective, multicenter analysis published recently, Azenha et al. reported only an incidence of 1.4% in a cohort of patients with clinical stage I, concluding that preoperative BI could be avoided in this subgroup [32]. In 2016, Lee et al. confirmed the low incidence of BMs in stage I in a retrospective analysis of 564 patients with SCC at an initial diagnosis of 0% in stage I [11]. Further studies identified for this review reported incidences of 0.5%, 0.3 (IA) to 3.8% (IB), 0%, and 2% in stage I NSCLC [27,33,35,43,47].

Despite those low incidences, Balekian et al. determined (based on the data available from the National Lung Screening Trial) that despite the lack of evidence for the need for BI in stage I, 12.5% of patients still underwent brain scans preoperatively [41]. None of those patients were diagnosed with BMs, and all of them went for curative-intent surgery [47]. The same overuse of BI in stage IA was reported by Milligan et al. [66]. This kind of over-screening in stage I has been shown to result in increased costs as well as therapy delays while only offering a marginal impact on patient management [64]. To make things worse, over-diagnosing with false positive rates in BI (MRI) in early-stage NSCLC was reported at 7.6% for stage I versus only a reasonable 1.4% for stage III patients [32].

According to “Choosing Wisely”, an initiative partnering with the Society of Thoracic Surgeons (STS), patients with suspected or biopsy-proven stage I NSCLC do not require preoperative BI unless neurologically symptomatic [67]. This is in line with the following statement published in the NICE guidelines: “Provide treatment without undue delay for people who have lung cancer that is suitable for radical treatment or chemotherapy, or who need radiotherapy or ablative treatment for relief of symptoms” [25].

The main reason for the debate on BI in early-stage NSCLC is silent/occult BMs. Zhuge et al. reported a BM rate of 5% in a study of about 3400 patients with NSCLC across all stages. Overall, 87.6% of the patients who had BMs were asymptomatic. According to these findings, the authors stated that routine neuroimaging could be beneficial in detecting occult BMs, especially in patients with T1c NSCLC. Notably, 105 of the investigated patients (3.1%) did not undergo surgery due to an unexpected BM found on MRI [36].

Silent BMs, in general, were found in approximately 30% of NSCLC patients across all stages [20]. Matys et al. found that more than 80% of BMs detected in potentially resectable NSCLC stage IA-IIIB patients were silent [38]. O’Dowd et al. reported, that diagnosed BMs in NSCLC were most likely to be those originally detected at an early stage (73% in stages I–II) and, for this reason, a preoperative neuroimaging should be performed [68]. Brockelsby et al. found a higher prevalence of BMs in their post-treatment cohort compared to metastases detected pre-surgery (7.9% vs. 2.1% in stage II, 7.8% vs. 5.2% in stage III) [31]. The burning question that remains is whether those BMs had been present prior to surgery with curative intent or not.

Even if the detection rate of BMs in early-stage NSCLC is relatively low, the ESMO and BTS guidelines as well as other authors like Hudson et al. recommend preoperative imaging in order to always offer the most appropriate management to all patients who are scheduled for surgery with curative intent [69]. Rami Porta et al. even recommended a regular BI prior to pulmonary resection except for patients with pure ground glass opacity (GGO), as did Cho et al. [27,59].

Shi et al. identified a significant percentage of patients (19.2%) who had isolated BM in the absence of nodal involvement. As patients with early-stage lung cancer and isolated BMs have better OS after resection of both the aforementioned, they raised the question of whether patients with resectable NSCLC, especially with high-risk adenocarcinoma or large cell carcinoma, should receive BI during staging [45].

Several authors have shown that the incidence of BMs after resection of NSCLC was higher than the incidence of BMs at diagnosis [31,42,68]. It was also reported that patients who developed BMs postoperatively had normal MRI brain preoperatively [42]. Therefore, the question arises of whether MRI brain should be incorporated into post-surgical follow-up in curatively resected NSCLC patients. The ACCP guidelines mention that a biannual follow-up MRI brain may detect early postoperative BMs, thereby providing an opportunity for radiotherapy or surgical treatment within a reasonable time [62]. Moreover, Ganau et al. showed that more accurate imaging would not only allow for radio-surgical options but also dictate the appropriateness of more tailored radio-surgical strategies including, e.g., multifocal, staged treatment with various sources of radiation therapy such as gamma knife, linear accelerators, etc. [70]. In times of availability of excellent local CNS therapy options, should we not give our patients the possibility to receive the best possible tailored treatment?

While offering the best possible tailored treatment, one should not forget the financial impact on the health care system. In a Canadian study by Vernon et al., additional staging MRI brain led to an increase in costs of about 45% per lung cancer case while only delivering additional staging information in 1.5% of the study population at the time of diagnosis. About a third of the preoperatively invested money for staging of any kind was invested in BI [42].

Brockelsby et al. 2023 performed a health economic analysis (in accordance with the previously performed analysis in the NICE guidelines), which concluded that BI was not cost-effective in stage II disease (ICERs GBP 50,023–GBP 115,785); however, BI remained cost-effective for stage III patients (ICERs GBP 17,000–GBP 22,173), with MRI being the most cost-effective strategy [31].

Gkogkozotou et al. reported, that BI in stage IA-IIIA NSCLC patients led to a reduction in avoidable thoracotomies, morbidity rates, and costs [40].

With the recent development of ongoing immunotherapy trials in the treatment of NSCLC and a more liberal approach to local treatment of brain-only metastases in NSCLC in a multidisciplinary setting, it is worth reconsidering the currently existing guidelines on BI in early-stage NSCLC [71]. Before deciding on BI in early-stage NSCLC, the benefits must certainly outweigh the risk of over or under imaging the brain.

In the setting of the biopsy-proven histology of primary NSCLC, patients with adenocarcinoma and/or known high-risk oncogenic driver mutations (EGFR, ALK), as well as patients of young age, might be considered to undergo BI due to the higher incidence of BM in this population [72]. In addition, Pedrosa et al. recently published a summary of the potential molecular signatures associated with the development of BMs in NSCLC (e.g., certain miRNAs, lncRNAs, and EGFR/KRAS&ALK) [73]. Consequently, all these identified criteria could help to identify patients at high risk of developing BMs secondary to NSCLC.

However, we are still far away from offering a robust risk prediction model to identify early-stage NSCLC patients at high risk of developing BMs.

### Limitations

This review has certain limitations. First, due to the inclusion of international studies, the heterogeneity in the varying applied national guidelines influencing the chosen staging process might have led to an overrepresentation of certain national guidelines. Secondly, the retrospective nature of the majority of the included studies demands more prospective data to achieve valid conclusions.

## 5. Conclusions

As summarized in the available guidelines on the staging of NSCLC patients, BI in stage I NSCLC patients is not recommended, especially in pure GGO and stage IA stages. Stage II NSCLC remains a matter of debate among guideline recommendations as well as among the identified and systematically reviewed studies. Furthermore, the avoidance of treatment delay while offering the most precise diagnostic staging for the most appropriate, tailored treatment was the main message of the included studies.

The focus of further investigations should lie on the identification of risk factors associated with BMs at the time of diagnosis for a more personalized and efficient screening, especially for subpopulations with assumed stages I and II NSCLC. Certain risk factors like the histological subtype and certain driver mutations are some of the preliminary steps toward being more efficient in preoperative CNS imaging.

In light of the above, our group suggests performing brain imaging starting from stage II and in symptomatic patients in any stage.

The identification of oncogenic driver mutations and the fact that, e.g., EGFR and ALK rearrangement were clinically significant risk factors for developing BM in NSCLC patients add to a more precise selection of patients suitable for preoperative BI [39]. Further studies are needed to receive a clear picture of the true prevalence of isolated BM among the staging subgroups in NSCLC and potentially rethink the clinical staging recommendations.

## Figures and Tables

**Table 1:** Existing guidelines on preoperative brain imaging in NSCLC patients.

Guideline, Year (Reference)	Imaging Modality	NSCLC Stages (UICC Eighth Ed.) with Screening Recommendation [Evidence Level]	Other Indications for Brain Imaging [Evidence Level]	Brain Imaging in Follow-Up
ACCP 2013 [20]	MRI or ce-CT if MRI not available	Stage III–IV, even if they have a negative clinical evaluation	Neurologically symptomatic patients.	No recommendation; biannual MRI mentioned
BTS 2010 [21]	MRI or ce-CT	Stage III, all stages when considered for curative therapy	Patients with features suggestive of intracranial pathology by an initial CT scan followed by MRI if normal or MRI as an initial test.	No recommendation
ESMO 2018, updated 2021 [22,23]	MRI preferred or ce-CT	Stage III	Stage I–II: Might be useful in patients considered for curative therapy. Most relevant in those patients with neurological symptoms or signs [IV, A].	No recommendation
NCCN 2018 [24]	MRI (if not possible, ce-CT)	Stage II–III	Stage IA: Not advised. Stage IB: Optional.	No recommendation
NICE 2019 [25,54]	MRI or ce-CT	Stage II–III, when treated with curative intent	Stage I: Not advised if asymptomatic. Patients with clinical features suggestive of intracranial pathology.	No recommendation

Abbreviations: ACCP, American College of Chest Physicians; BTS: British Thoracic Society; ESMO: European Society for Medical Oncology; NCCN: National Comprehensive Cancer Network; NICE: National Institute for Health and Care Excellence; NSCLC: non-small cell lung carcinoma; MRI: magnetic resonance imaging; ce-CT: contrast-enhanced computed tomography. (Evidence levels differ according to the respective guidelines).

## Data Availability

The studies and guidelines on which this systematic review are based can be obtained from the corresponding author upon reasonable request, provided that the copyrights of the respective authors are respected.

## Supplementary Materials

The following supporting information can be downloaded at: https://www.mdpi.com/article/10.3390/jcm14030708/s1, Table S1: 25 studies were finally included in this systematic review.

## Abbreviations

ACCP	American College of Chest Physicians
BI	brain imaging
BM	brain metastases
ce-MRI	contrast-enhanced magnetic resonance tomography
ce-CT	contrast-enhanced computed tomography
CNS	Central Nervous System
CT	computed tomography
EORTC	European Organization for Research and Treatment of Cancer
ESMO	European Society for Medical Oncology
FDG	F-fluoro-2-deoxy-D-glucose
GGO	ground glass opacities
MRI	magnetic resonance imaging
NCCN	National Comprehensive Cancer Network
NICE	National Institute for Health and Care Excellence
NSCLC	non-small cell lung cancer
OS	overall survival
PET	positron-emission computed tomography
QoL	quality of life
SCC	squamous cell carcinoma
STS	Society of Thoracic Surgeons
TNM	Tumor Node Metastasis
UICC	Union for International Cancer Control

## References

[1] Torre L.A., Bray F., Siegel R.L., Ferlay J., Lortet-Tieulent J., Jemal A. (2015). Global cancer statistics, 2012. CA Cancer J. Clin..

[2] BWS CPW World Cancer Report 2014. https://publications.iarc.fr/Non-Series-Publications/World-Cancer-Reports/World-Cancer-Report-2014.

[3] Fitzmaurice C., Dicker D., Pain A., Hamavid H., Moradi-Lakeh M., MacIntyre M.F., Allen C., Hansen G., Woodbrook R., Global Burden of Disease Cancer Collaboration (2015). The Global Burden of Cancer 2013. JAMA Oncol..

[4] Sánchez de Cos J., Sojo González M.A., Montero M.V., Pérez Calvo M.C., Vicente M.J., Valle M.H. (2009). Non-small cell lung cancer and silent brain metastasis. Survival and prognostic factors. Lung Cancer.

[5] Nayak L., Lee E.Q., Wen P.Y. (2012). Epidemiology of brain metastases. Curr. Oncol. Rep..

[6] Cagney D.N., Martin A.M., Catalano P.J., Redig A.J., Lin N.U., Lee E.Q., Wen P.Y., Dunn I.F., Bi W.L., Weiss S.E. (2017). Incidence and prognosis of patients with brain metastases at diagnosis of systemic malignancy: A population-based study. Neuro Oncol..

[7] Schouten L.J., Rutten J., Huveneers H.A., Twijnstra A. (2002). Incidence of brain metastases in a cohort of patients with carcinoma of the breast, colon, kidney, and lung and melanoma. Cancer.

[8] Barnholtz-Sloan J.S., Sloan A.E., Davis F.G., Vigneau F.D., Lai P., Sawaya R.E. (1973). Incidence proportions of brain metastases in patients diagnosed (1973 to 2001) in the Metropolitan Detroit Cancer Surveillance System. J. Clin. Oncol..

[9] Moro-Sibilot D., Smit E., de Castro Carpeño J., Lesniewski-Kmak K., Aerts J.G., Villatoro R., Kraaij K., Nacerddine K., Dyachkova Y., Smith K.T. (2015). Non-small cell lung cancer patients with brain metastases treated with first-line platinum-doublet chemotherapy: Analysis from the European FRAME study. Lung Cancer.

[10] Peters S., Bexelius C., Munk V., Leighl N. (2016). The impact of brain metastasis on quality of life, resource utilization and survival in patients with non-small-cell lung cancer. Cancer Treat. Rev..

[11] Lee H., Jeong S.H., Jeong B.H., Park H.Y., Lee K.J., Um S.W., Kwon O.J., Kim H. (2016). Incidence of Brain Metastasis at the Initial Diagnosis of Lung Squamous Cell Carcinoma on the Basis of Stage, Excluding Brain Metastasis. J. Thorac. Oncol..

[12] Pichert M.D., Canavan M.E., Maduka R.C., Li A.X., Ermer T., Zhan P.L., Kaminski M., Udelsman B.V., Blasberg J.D., Mase V.J. (2022). Revisiting Indications for Brain Imaging During the Clinical Staging Evaluation of Lung Cancer. JTO Clin. Res. Rep..

[13] Kim M., Suh C.H., Lee S.M., Park J.E., Kim H.C., Kim S.O., Aizer A.A., Yanagihara T.K., Bai H.X., Guenette J.P. (2021). Development of Brain Metastases in Patients with Non-Small Cell Lung Cancer and No Brain Metastases at Initial Staging Evaluation: Cumulative Incidence and Risk Factor Analysis. AJR Am. J. Roentgenol..

[14] Hudson B.J., Crawford M.B., Curtin J.J. (2015). Brain imaging in lung cancer patients without symptoms of brain metastases: A national survey of current practice in England. Clin. Radiol..

[15] Yokoi K., Kamiya N., Matsuguma H., Machida S., Hirose T., Mori K., Tominaga K. (1999). Detection of brain metastasis in potentially operable non-small cell lung cancer: A comparison of CT and MRI. Chest.

[16] Balasubramanian S.K., Sharma M., Venur V.A., Schmitt P., Kotecha R., Chao S.T., Suh J.H., Angelov L., Mohammadi A.M., Vogelbaum M.A. (2020). Impact of EGFR mutation and ALK rearrangement on the outcomes of non-small cell lung cancer patients with brain metastasis. Neuro Oncol..

[17] Billing P.S., Miller D.L., Allen M.S., Deschamps C., Trastek V.F., Pairolero P.C. (2001). Surgical treatment of primary lung cancer with synchronous brain metastases. J. Thorac. Cardiovasc. Surg..

[18] Patchell R.A., Tibbs P.A., Walsh J.W., Dempsey R.J., Maruyama Y., Kryscio R.J., Markesbery W.R., Macdonald J.S., Young B. (1990). A randomized trial of surgery in the treatment of single metastases to the brain. N. Engl. J. Med..

[19] Noordijk E.M., Vecht C.J., Haaxma-Reiche H., Padberg G.W., Voormolen J.H., Hoekstra F.H., Tans J.T., Lambooij N., Metsaars J.A., Wattendorff A.R. (1994). The choice of treatment of single brain metastasis should be based on extracranial tumor activity and age. Int. J. Radiat. Oncol. Biol. Phys..

[20] Silvestri G.A., Gonzalez A.V., Jantz M.A., Margolis M.L., Gould M.K., Tanoue L.T., Harris L.J., Detterbeck F.C. (2013). Methods for staging non-small cell lung cancer: Diagnosis and management of lung cancer, 3rd ed: American College of Chest Physicians evidence-based clinical practice guidelines. Chest.

[21] Lim E., Baldwin D., Beckles M., Duffy J., Entwisle J., Faivre-Finn C., Kerr K., Macfie A., McGuigan J., Padley S. (2010). Guidelines on the radical management of patients with lung cancer. Thorax.

[22] Postmus P.E., Kerr K.M., Oudkerk M., Senan S., Waller D.A., Vansteenkiste J., Escriu C., Peters S. (2017). ESMO Guidelines Committee. Early and locally advanced non-small-cell lung cancer (NSCLC): ESMO Clinical Practice Guidelines for diagnosis, treatment and follow-up. Ann. Oncol..

[23] Early and Locally Advanced Non-Small-Cell Lung Cancer. https://www.esmo.org/guidelines/guidelines-by-topic/esmo-clinical-practice-guidelines-lung-and-chest-tumours/early-stage-and-locally-advanced-non-metastatic-non-small-cell-lung-cancer/eupdate-early-and-locally-advanced-non-small-cell-lung-cancer-nsclc-treatment-recommendations2.

[24] Ettinger D.S., Aisner D.L., Wood D.E., Akerley W., Bauman J., Chang J.Y., Chirieac L.R., D’Amico T.A., Dilling T.J., Dobelbower M. (2018). NCCN Guidelines Insights: Non-Small Cell Lung Cancer, Version 5.2018. J. Natl. Compr. Cancer Netw..

[25] Baldwin D.R., White B., Schmidt-Hansen M., Champion A.R., Melder A.M., Guideline Development Group (2011). Diagnosis and treatment of lung cancer: Summary of updated NICE guidance. BMJ.

[26] Villano J.L., Durbin E.B., Normandeau C., Thakkar J.P., Moirangthem V., Davis F.G. (2015). Incidence of brain metastasis at initial presentation of lung cancer. Neuro Oncol..

[27] Cho H., Lee H.Y., Kim J., Kim H.K., Choi J.Y., Um S.W., Lee K.S. (2015). Pure ground glass nodular adenocarcinomas: Are preoperative positron emission tomography/computed tomography and brain magnetic resonance imaging useful or necessary?. J. Thorac. Cardiovasc. Surg..

[28] Nam J.G., Hong H., Choi S.H., Park C.M., Goo J.M., Kim Y.T., Kim H. (2022). No Prognostic Impact of Staging Brain MRI in Patients with Stage IA Non-Small Cell Lung Cancer. Radiology.

[29] Page M.J., McKenzie J.E., Bossuyt P.M., Boutron I., Hoffmann T.C., Mulrow C.D., Shamseer L., Tetzlaff J.M., Akl E.A., Brennan S.E. (2021). The PRISMA 2020 statement: An updated guideline for reporting systematic reviews. BMJ.

[30] McGuinness L.A., Higgins J.P.T. (2021). Risk-of-bias VISualization (robvis): An R package and Shiny web app for visualizing risk-of-bias assessments. Res. Synth. Methods.

[31] Brockelsby C., Maconachie R., Navani N., Prendecki R., Randles V., King J., Dildar B., Lee X., Nagarajan T., Rice M. (2023). Brain imaging in lung cancer staging: A real-world, multi-centre study of prevalence of brain metastases, impact on treatment and re-modelling of the NICE health economic analysis. Lung Cancer.

[32] Azenha L.F., Bertoglio P., Kestenholz P., Gonzalez M., Pal M., Krueger T., Redwan B., Koesek V., Masri E.A., Miyazaki T. (2022). Role of Pre-Operative Brain Imaging in Patients with NSCLC Stage I: A Retrospective, Multicenter Analysis. Cancers.

[33] Saito G., Kono M., Koyanagi Y., Miyashita K., Tsutsumi A., Kobayashi T., Miki Y., Hashimoto D., Nakamura T., Nozue M. (2021). Significance of Brain Imaging for Staging in Patients With Clinical Stage T1-2 N0 Non-Small-Cell Lung Cancer on Positron Emission Tomography/Computed Tomography. Clin. Lung Cancer.

[34] Naresh G., Malik P.S., Khurana S., Pushpam D., Sharma V., Yadav M., Jain D., Pathy S. (2021). Assessment of Brain Metastasis at Diagnosis in Non–Small-Cell Lung Cancer: A Prospective Observational Study from North India. JCO Glob. Oncol..

[35] Kim M., Suh C.H., Lee S.M., Kim H.C., Aizer A.A., Yanagihara T.K., Bai H.X., Guenette J.P., Huang R.Y., Kim H.S. (2020). Diagnostic Yield of Staging Brain MRI in Patients with Newly Diagnosed Non-Small Cell Lung Cancer. Radiology.

[36] Zhuge L., Huang Y., Wang S., Xie J., Huang B., Zheng D., Zheng S., Zhao Y., Mao H., Wilson D.O. (2019). Preoperative brain MRI for clinical stage IA lung cancer: Is routine scanning rational?. J. Cancer Res. Clin. Oncol..

[37] Schoenmaekers J., Hofman P., Bootsma G., Westenend M., de Booij M., Schreurs W., Houben R., De Ruysscher D., Dingemans A.M., Hendriks L.E. (2019). Screening for brain metastases in patients with stage III non-small-cell lung cancer, magnetic resonance imaging or computed tomography? A prospective study. Eur. J. Cancer.

[38] Matys T., Drury R., David S., Rassl D.M., Qian W., Rintoul R.C., Screaton N.J. (2018). Routine preoperative brain CT in resectable non-small cell lung cancer—Ten years experience from a tertiary UK thoracic center. Lung Cancer.

[39] Ando T., Kage H., Saito M., Amano Y., Goto Y., Nakajima J., Nagase T. (2018). Early stage non-small cell lung cancer patients need brain imaging regardless of symptoms. Int. J. Clin. Oncol..

[40] Gkogkozotou V.I., Gkiozos I.C., Charpidou A.G., Kotteas E.A., Boura P.G., Tsagouli S.N., Syrigos K.N. (2018). PET/CT and brain MRI role in staging NSCLC: Prospective assessment of the accuracy, reliability and cost-effectiveness. Lung Cancer Manag..

[41] Balekian A.A., Fisher J.M., Gould M.K. (2016). Brain Imaging for Staging of Patients with Clinical Stage IA Non-small Cell Lung Cancer in the National Lung Screening Trial: Adherence with Recommendations From the Choosing Wisely Campaign. Chest.

[42] Vernon J., Andruszkiewicz N., Schneider L., Schieman C., Finley C.J., Shargall Y., Fahim C., Farrokhyar F., Hanna W.C. (2016). Comprehensive Clinical Staging for Resectable Lung Cancer: Clinicopathological Correlations and the Role of Brain MRI. J. Thorac. Oncol..

[43] Na I.I., Lee T.H., Choe D.H., Cheon G.J., Kim C.H., Koh J.S., Baek H., Ryoo B.Y., Yang S.H., Lee J.C. (2008). A diagnostic model to detect silent brain metastases in patients with non-small cell lung cancer. Eur. J. Cancer.

[44] Park H.Y., Kim Y.H., Kim H., Koh W.J., Suh G.Y., Chung M.P., Kwon O.J. (2007). Routine screening by brain magnetic resonance imaging decreased the brain metastasis rate following surgery for lung adenocarcinoma. Lung Cancer.

[45] Shi A.A., Digumarthy S.R., Temel J.S., Halpern E.F., Kuester L.B., Aquino S.L. (2006). Does initial staging or tumor histology better identify asymptomatic brain metastases in patients with non-small cell lung cancer?. J. Thorac. Oncol..

[46] Kim S.Y., Kim J.S., Park H.S., Cho M.J., Kim J.O., Kim J.W., Song C.J., Lim S.P., Jung S.S. (2005). Screening of brain metastasis with limited magnetic resonance imaging (MRI): Clinical implications of using limited brain MRI during initial staging for non-small cell lung cancer patients. J. Korean Med. Sci..

[47] Yohena T., Yoshino I., Kitajima M., Uehara T., Kanematsu T., Teruya T., Ikeda J., Ichinose Y. (2004). Necessity of preoperative screening for brain metastasis in non-small cell lung cancer patients without lymph node metastasis. Ann. Thorac. Cardiovasc. Surg..

[48] Hochstenbag M.M., Twijnstra A., Hofman P., Wouters E.F., ten Velde G.P. (2003). MR-imaging of the brain of neurologic asymptomatic patients with large cell or adenocarcinoma of the lung. Does it influence prognosis and treatment?. Lung Cancer.

[49] Tanaka K., Kubota K., Kodama T., Nagai K., Nishiwaki Y. (1999). Extrathoracic staging is not necessary for non-small-cell lung cancer with clinical stage T1-2 N0. Ann. Thorac. Surg..

[50] Earnest F., Ryu J.H., Miller G.M., Luetmer P.H., Forstrom L.A., Burnett O.L., Rowland C.M., Swensen S.J., Midthun D.E. (1999). Suspected Non-Small Cell Lung Cancer: Incidence of Occult Brain and Skeletal Metastases and Effectiveness of Imaging for Detection—Pilot Study. Radiology.

[51] Purandare N.C., Rangarajan V. (2015). Imaging of lung cancer: Implications on staging and management. Indian. J. Radiol. Imaging.

[52] Planchard D., Popat S., Kerr K., Novello S., Smit E.F., Faivre-Finn C., Mok T.S., Reck M., Van Schil P.E., Hellmann M.D. (2018). Metastatic non-small cell lung cancer: ESMO Clinical Practice Guidelines for diagnosis, treatment and follow-up. Ann. Oncol..

[53] Kuhn M.J., Hammer G.M., Swenson L.C., Youssef H.T., Gleason T.J. (1994). MRI evaluation of “solitary” brain metastases with triple-dose gadoteridol: Comparison with contrast-enhanced CT and conventional-dose gadopentetate dimeglumine MRI studies in the same patients. Comput. Med. Imaging Graph..

[54] Collins L.G., Haines C., Perkel R., Enck R.E. (2007). Lung cancer: Diagnosis and management. Am. Fam. Physician.

[55] Colice G.L., Birkmeyer J.D., Black W.C., Littenberg B., Silvestri G. (1995). Cost-effectiveness of head CT in patients with lung cancer without clinical evidence of metastases. Chest.

[56] De Wever W., Bruyeer E., Demaerel P., Wilms G., Coolen J., Verschakelen J. (2010). Staging of lung cancer. Do we need a diagnostic CT of the brain after an integrated PET/CT for the detection of brain metastases?. J. Belg. Soc. Radiol..

[57] Nosotti M., Castellani M., Longari V., Chella B., Baisi A., Rosso L., Santambrogio L. (2008). Staging non-small lung cancer with positron emission tomography: Diagnostic value, impact on patient management, and cost-effectiveness. Int. Surg..

[58] Lee S.M., Goo J.M., Park C.M., Yoon S.H., Paeng J.C., Cheon G.J., Kim Y.T., Park Y.S. (2016). Preoperative staging of non-small cell lung cancer: Prospective comparison of PET/MR and PET/CT. Eur. Radiol..

[59] Rami-Porta R., Call S., Dooms C., Obiols C., Sánchez M., Travis W.D., Vollmer I. (2018). Lung cancer staging: A concise update. Eur. Respir. J..

[60] Yi C.A., Lee K.S., Lee H.Y., Kim S., Kwon O.J., Kim H., Choi J.Y., Kim B.T., Hwang H.S., Shim Y.M. (2013). Coregistered whole body magnetic resonance imaging-positron emission tomography (MRI-PET) versus PET-computed tomography plus brain MRI in staging resectable lung cancer: Comparisons of clinical effectiveness in a randomized trial. Cancer.

[61] Deuschl C., Nensa F., Grueneisen J., Poeppel T.D., Sawicki L.M., Heusch P., Gramsch C., Mönninghoff C., Quick H.H., Forsting M. (2017). Diagnostic impact of integrated 18F-FDG PET/MRI in cerebral staging of patients with non-small cell lung cancer. Acta Radiol..

[62] Nishikawa T., Ueba T., Kawashima M., Kajiwara M., Iwata R., Kato M., Miyamatsu N., Yamashita K. (2010). Early detection of metachronous brain metastases by biannual brain MRI follow-up may provide patients with non-small cell lung cancer with more opportunities to have radiosurgery. Clin. Neurol. Neurosurg..

[63] Goldstraw P., Chansky K., Crowley J., Rami-Porta R., Asamura H., Eberhardt W.E., Nicholson A.G., Groome P., Mitchell A., Bolejack V. (2016). The IASLC Lung Cancer Staging Project: Proposals for Revision of the TNM Stage Groupings in the Forthcoming (Eighth) Edition of the TNM Classification for Lung Cancer. J. Thorac. Oncol..

[64] Levy A., Faivre-Finn C., Hasan B., De Maio E., Berghoff A.S., Girard N., Greillier L., Lantuéjoul S., O’Brien M., Reck M. (2018). Diversity of brain metastases screening and management in non-small cell lung cancer in Europe: Results of the European Organisation for Research and Treatment of Cancer Lung Cancer Group survey. Eur. J. Cancer.

[65] Abdel-Rahman O. (2019). Is routine baseline brain imaging needed for all newly diagnosed non-small-cell lung cancer patients?. J. Comp. Eff. Res..

[66] Milligan M.G., Cronin A.M., Colson Y., Kehl K., Yeboa D.N., Schrag D., Chen A.B. (2020). Overuse of Diagnostic Brain Imaging Among Patients With Stage IA Non-Small Cell Lung Cancer. J. Natl. Compr. Cancer Netw..

[67] Wood D.E., Mitchell J.D., Schmitz D.S., Grondin S.C., Ikonomidis J.S., Bakaeen F.G., Merritt R.E., Meyer D.M., Moffatt-Bruce S.D., Reece T.B. (2013). Choosing wisely: Cardiothoracic surgeons partnering with patients to make good health care decisions. Ann. Thorac. Surg..

[68] O’Dowd E.L., Kumaran M., Anwar S., Palomo B., Baldwin D.R. (2014). Brain metastases following radical surgical treatment of non-small cell lung cancer: Is preoperative brain imaging important?. Lung Cancer.

[69] Hudson Z., Internullo E., Edey A., Laurence I., Bianchi D., Addeo A. (2017). Brain imaging before primary lung cancer resection: A controversial topic. Ecancermedicalscience.

[70] Ganau M., Foroni R.I., Gerosa M., Zivelonghi E., Longhi M., Nicolato A. (2014). Radiosurgical options in neuro-oncology: A review on current tenets and future opportunities. Part I: Therapeutic strategies. Tumori J..

[71] Felip E., Altorki N., Zhou C., Csőszi T., Vynnychenko I., Goloborodko O., Luft A., Akopov A., Martinez-Marti A., Kenmotsu H. (2021). Adjuvant atezolizumab after adjuvant chemotherapy in resected stage IB-IIIA non-small-cell lung cancer (IMpower010): A randomised, multicentre, open-label, phase 3 trial. Lancet.

[72] Mujoomdar A., Austin J.H., Malhotra R., Powell C.A., Pearson G.D., Shiau M.C., Raftopoulos H. (2007). Clinical predictors of metastatic disease to the brain from non-small cell lung carcinoma: Primary tumor size, cell type, and lymph node metastases. Radiology.

[73] Pedrosa R.M.S.M., Mustafa D.A.M., Aerts J.G.J.V., Kros J.M. (2018). Potential Molecular Signatures Predictive of Lung Cancer Brain Metastasis. Front. Oncol..

