# Supplementary material for: Brain Imaging in Patients with Non-Small Cell Lung Cancer—A Systematic Review
Source: J Clin Med. 2025 Jan 22;14(3):708. doi: 10.3390/jcm14030708 (PMC11818832; doi:10.3390/jcm14030708)
Supplement: Supplementary file 1 [file jcm-14-00708-s001.zip › jcm-3399455-supplementary.pdf]

Supplementary material Table S1. 25 studies were finally included in this systematic review. An overview of all studies is demonstrated in Table 1. Studies were sorted in descending order by year of publication. For more detailed information the respective reference is mentioned in Table 1. [11,12,15,27,28,31–50].

**Author [Reference]**	**Year Publication (recruitement)**	**Study design/ database/ country**	**Number (n)**	**Sex male (%)**	**Stage (TNM edition)**	**Exclusion criteria**	**Incidence of BM at staging (%)**	**BI frequency (%)**	**Imaging modality (%)**	**Histology of primary cancer (%)**	**Histology of BM**	**Up-Staging Stage/%**	**Conclusion**
**Brockelsby et al. [31]**	2023 (01/2018 - 12/2018)	Retrospective Multi-centre<br><br>UK	444	-	II-III (8th edition)	Neurological symptoms	3.65%<br><br>II: 2.1%<br>III: 5.2%	II: 29%<br><br>III: 68%	MRI	Stage II AC 57%<br>Stage III AC 41%	Not reported	(7/10) 70% in stage III had treatment changed to palliative	Recommendation for BI prior to curative-intent treatment in stage III, questionable is the cost-effectiveness of CT BI prior to curative-intent treatment in stage II
**Pichert et al. [12]**	2022 (2016-2017)	Retrospective<br><br>US-National Cancer Database<br><br>USA	149 958	-	I-III<br><br>BM limited stage IV<br><br>(7th edition)	Metastases in locations other than the brain	4.6%<br><br>IA: 1.7%<br>IB: 3.7%<br>IIA: 6.4%<br>IIB: 5.8%<br>IIIA: 6.7%<br>IIIB: 5%	100%	Not reported	NOS	Not reported	none	Evidence of clear differences in the prevalence of BM in NSCLC across staging. It is less clear whether these differences are of sufficient magnitude to differentiate reasonable from unreasonable indications for BI.
**Nam et al. [28]**	2022 (02/2009 - 03/2016)	Retrospective single centre<br><br>Korea	331	51%	IA (8th edition)<br><br>cT1mi 2%<br>cT1a 6%<br>cT1b 46%<br>cT1c 46%	Pure GGO, metachronous or synchronous lung cancer, SCLC, low-grade malignancies, sub-lobar resection	4.8%	100%	MRI	AC 83%<br>SCC 14%<br>LCC/Other 3%	Not reported	0.3%	No prognostic impact of BI in stage IA NSCLC.
**Azenha et al. [32]**	2022 (01/2019 – 12/2020)	Retrospective Multi-centre<br><br>Switzerland	577	55.5%	I (8th edition)	Prior history of lung cancer, synchronous or metachronous disease, previous brain malignancies,	1.4%	100%	ce-MRI 83.2%<br><br>ce-CT 16.8%	AC 69.3%<br>SCC 23.4%<br>Other 7.3%	AC 100%	none	Avoid BI in stage I.

						neurological symptoms							
**Naresh et al. [34]**	2021<br>(01/2018 - 10/2019)	Prospective<br>Single centre<br><br>India	496	75%	I-IV<br><br>IV: 74%<br>III: 21%<br><br>(8th edition)	No BI<br>No tissue diagnosis	21%<br><br>I-IIIA: 2.4%<br>IIIB-IIIC: 5%<br>IV: 13.5%	100%	ce-CT/MRI	AC 64%<br>SCC 27%<br>Other 9%	AC 28%<br>NOS 21%<br>SCC 4%	7%	BI with ce-CT leads to detection of BM in up to 21%. The disease is upstaged in 7% after BI. molGPA help in appropriate risk stratification for a better patient management.
**Saito et al. [33]**	2021<br>(2008 – 2016)	Retrospective<br>Single centre<br><br>Japan	466	58%	I-IIA<br>T1-2 N0<br>(8th edition)	No HRCT, no PET-CT, no MRI brain	0.6%<br><br>T1: 0.5%<br>T2: 3%	100%	ce-MRI	AC 81%<br>SCC 14%<br>Other 5%	Not reported	Not reported	Routine BI is unnecessary in patients with early-stage NSCLC, assessed with a staging protocol including PET-CT.
**Kim et al. [35]**	2020<br>(11/2017 - 10/2018)	Retrospective<br>Single centre<br><br>Korea	1712	60.5%	I-IV<br>(8th edition)<br><br>I: 801<br>II: 171<br>III: 342<br>IV: 398	Malignancy other than lung cancer, without staging brain MRI, without CT chest report and with clinical staging Tis	11.9%<br><br>I: 1.1%<br>IA: 0.3%<br>IB: 3.8%<br>II: 4.7%<br>III: 13.2%<br>IV: 35.4%	100%	ce-MRI	AC 75.8%<br>SCC 20.7%	Not reported	Not reported	MRI of the brain has low diagnostic yield in stage IA NSCLC. There is higher diagnostic yield in NSCLC stage IB and EGFR-mutated patients.
**Zhuge et al. [36]**	2019<br>(2007-2016)	Retrospective<br>Single centre<br><br>China	3392	56%	I-IV<br>(8th edition)<br><br>IA : 47.0%<br>*T1a 11.5%*<br>*T1b 20.3%*<br>*T1c 15.2%*<br><br>IB 7.4%<br>II 15.7%<br>III 22.9%<br>IV 7.0%	Age<18y	5%<br><br>IA: 0.7%<br>IB: 2.0%<br>II: 3.6%<br>III: 8.8%<br>IV: 26.2%	100%	MRI	AC 70%<br>SCC 18%<br>Other 12%	AC 69%<br>SCC 5%<br>Other 26%	Cancellation of surgery or alteration of treatment in 3.1%	In stage IA, preoperative brain MRI not recommended, but it may potentially be beneficial in solid T1c cancers.
**Schoenmaekers et al. [37]**	2019<br>(12/2012 – 10/2017)	Prospective<br>Multicentre<br><br>Netherlands	149	-	III-IV<br>(7th edition)	BM detected on ce-CT at staging<br><br>2nd primary cancer within 2y of stage III NSCLC diagnosis, no BI, mixed histology	4.7%	100%	MRI	AC 45%<br>SCC 38.3%<br>LCC 2.7%<br>NOS 12.8&<br>LCNEC 1.3%	Not reported	4.7% from stage III-IV	Screening for BM is mandatory in the work-up of stage III NSCLC patients, and MRI is superior to a dedicated brain ce-CT.
**Matys et al. [38]**	2018	Retrospective<br>Single centre	1074	59%	IA-IIIA<br>(7th edition)	Stage IIIB-V	2.1%	100%	ce-CT	AC 40%<br>SCC 38%	Not reported	1.6% did not have surgery	Except for stage IA, in which the detection rate

		UK					IA: 0.7%<br>IB: 1.6%<br>IIA: 3%<br>IIB: 4.3%<br>IIIA: 2.6%			NOS 18%<br>Other+non-invasive AC 4%		due to upstaging	is very low, ce-CT could be useful in routine NSCLC staging.
**Ando et al. [39]**	2018<br>(01/2012 - 04/2016)	Retrospective<br>Single centre<br><br>Japan	124	37%	IVb<br>(7th edition)	No staging according to TNM possible, stage IVa	37%	100%	MRI or CT	AC 76%<br>SCC 7%<br>NOS 17%	Not reported	Not reported	Routine BI seems warranted in all NSCLC patients, especially in regions with higher frequency of EGFR mutations.
**Gkogkozotou et al. [40]**	2018<br>(12/2014 to 11/2016)	Retrospective<br>Single centre<br><br>Greece	30	83.3%	IA -IIIA	Bone metastases, previous resection/therapy of NSCLC, SCLC, stage>IIIA	0%	100%	MRI	SCC 70%<br>AC 26.7%<br>Other 3.3%	Not reported	Not reported	PET-CT and brain MRI combined are reliable for correct staging, reducing avoidable thoracotomies, morbidity and costs.
**Balekian et al. [41]**	2016	Retrospective<br>Multicentre<br><br>National Lung Screening Trial<br><br>USA	643	54%	IA<br>(7th edition)	SCLC, T >T1, missing clinical N-stage, N>N0, M1	0%	12 %	CT or MRI not further specified	AC 49%<br>SCC 27%<br>Other 23%	-	1.1%, not due to BM	No BI in asymptomatic patients with stage IA.
**Vernon et al. [42]**	2016<br>(01/2012 – 06/2014)	Retrospective<br>Single centre<br><br>Canada	315	44.4%	I-IV<br>(7th edition)	Patients with non-resectable NSCLC	1.5%<br><br>3.5% incl. postop FU	87%	MRI	SCC 27.6%<br>AC 55.9%<br>Other 16.5%	-	none	Results of staging with ce-CT chest, PET-CT with/without MRI were identical 98.8%. Questionable is the value of routine preoperative brain MRI in this population owing to its high costs and low yield.
**Lee et al. [11]**	2016<br>(01/2012 - 12/2013)	Retrospective<br>Single centre<br><br>Korea	564	93.3%	I-IV<br>(7th edition)	Lung cancer other than SCC	5%<br><br>IA: 0%<br>IB: 0%<br>IIA: 2.4%<br>IIB: 3.8%<br>IIIA: 1.9%<br>IIIB: 5.9 %<br>IV: 18.2%	100%	MRI	SCC 100%	-	Upstaging from potentially operable stage (IA-IIIA to stage IV in 0.5%	Routine brain MRI screening in patients with stage II to IV lung SCC might help to evaluate asymptomatic BM. No screening in stage I.

**Cho et al. [27]**	2015<br>(10/2003<br>-<br>02/2014)	Retrospective<br>Single centre<br><br>Korea	164	45%	IA1<br>(7th edition)	NSCLC with solid component	0%	66.5%	MRI	GGO AC 100%	-	-	PET-CT and brain MRI is not necessary in the staging of pure GGO AC
**Na et al. [43]**	2008<br>(04/2003<br>–<br>04/2007)	Retrospective<br>Single centre<br><br>Korea	433	77%	I-IV<br>(6th edition)	Sarcomatoid carcinoma, adenosquamous cell carcinoma	7%<br><br>I : 2%<br>II : 5%<br>III : 6%	100%	Ce-MRI	SCC 47%<br>AC 41%<br>LCC 2%<br>NOS 10%	-	-	Identified risk factors may be useful to identify patients at high risk of silent BM.
**Park et al. [44]**	2007<br>(01/1995<br>–<br>12/2000)	Retrospective<br>Single centre<br><br>Korea	83	55%	IA-IIIB<br>(6th edition)<br><br>IA29%<br>IB 33%<br>IIA 2%<br>IIB 16%<br>IIIA 20%<br>IIIB 0%	NSCLC other than AC	0%	100%	Ce-MRI	AC 100%	Not reported	Not reported	Preoperative MR screening of the brain can help early detection of BM in patients with AC prior to surgical resection and lead to increase postoperative survival
**Shi et al. [45]**	2006<br>(01/1996<br>–<br>03/2003)	Retrospective<br>Single centre<br><br>USA	809<br><br>120<br>Sympt.<br>Group 1<br><br>61<br>Asympt.<br>Group 2	 <br>49%<br>Group<br>1<br><br>50.8%<br>Group<br>2	I-IV<br>(6th edition)	No BI	22.4%<br><br>14.8%<br>Group 1<br><br>7.5%<br>Group 2	100%	MRI or CT	AC 58.6%<br>LCC 17.7%<br>SCC 9.9%	Not reported	Not reported	High number of asymptomatic BM in early-stage NSCLC, incidence higher in AC and LCC
**Kim et al. [46]**	2005<br>(Study group<br>05/2001<br>–<br>04/2002)<br><br>(Control group<br>05/2000<br>–<br>04/2001)	Retrospective<br>Single centre<br><br>Korea	183<br>Study group<br><br>131<br>Sympt.<br>group	76%<br>Study group<br><br>80%<br>Sympt.<br>group	I-IV<br>(6th edition)	Pathology other than NSCLC	20.8%<br>Study group<br><br>4.6%<br>Sympt.<br>group	100%<br>Study group<br><br>4.6%<br>Sympt.<br>group	Limited MRI<br>*(see reference for specification)*	Not reported	-	13.5% overall<br><br>15.9% of patients initially thought to be resectable	Limited brain MRI appears to be a useful and cost-effective means to detect BM at the time of initial staging NSCLC.
**Yohena et al. [47]**	2004<br>(04/1996<br>–<br>12/1998)	Retrospective<br>Single centre<br><br>Japan	141	69.5%	I-III<br>(6th edition)	Patients not considered for surgery in curative intent (T4, cN3,	2.1%<br><br>N0 : 0%<br>N1 : 5.2%<br>N2 : 4.7%	100%	MRI	AC 57.4%<br>SCC 29.8%<br>SCLC 6.4%<br>LCC 2.8%	AC 100%	none	Resectable NSCLC, no brain MRI indicated if no distant lesions and neurologically asymptomatic

						cM1, neurological symptoms)							
**Hochstenbag et al. [48]**	2003<br>(01/1996 to 01/2000)	Retrospective<br>Single centre<br><br>Netherlands	91	76%	I-IV<br>(6th edition)	Neurologically symptomatic	14%<br><br>I: 0%<br>II: 8.3%<br>IIIA: 21%<br>IIIB: 10.5%<br>IV: 28.6%	100%	Ce-MRI	LCC 55%<br>AC 45%	-	8%<br><br>I/II: 3%<br>IIIA: 21%<br>IIIB: 11%	MRI brain recommended in staging for stage III AC and LCC
**Tanaka et al. [49]**	1999<br>(01/1982 - 05/1996)	Retrospective<br>Single centre<br><br>Japan	754	61.1%	I-IIA<br>(T1-2 N0)<br>(6th edition)	peripheral LN metastases and/or metastases in contralateral lung	T1N0: 0.7%<br>T2N0: 2.1%	100%	Ce-CT or ce-MRI	AC 66.2%<br>SCC 27.4%<br>LCC 1.6%<br>Other 4.8%	-	1.3%<br><br>2.5% due to bone and liver metastases	Considering the cost and time savings, staging procedures are not warranted for patients with NSCLC stage T1–2 N0 with negative clinical evaluations
**Yokoi et al. [15]**	1999<br>(01/1989 – 12/1995)	Prospective<br>Single centre<br><br>Japan	332	68%	I-IIIB<br>(6th edition)	Neurologically symptomatic	MRI: 3.4%<br>CT: 0.6%	MRI: 53.3%<br>CT: 46.7%	Ce-CT or ce-MRI	AC 57.5%<br>SCC 37.3%<br>Other 5.1%	AC 61%<br>SCC 26%<br>Other 3%	-	Preoperative evaluation and FU with MRI could facilitate early detection of BM in patients with potentially operable NSCLC
**Earnest et al. [50]**	1999	Prospective<br>Single centre<br><br>USA	27	76%	I-IV<br>(6th edition)	T1 N0 M0	22%	100%	Ce-MRI	SCC 48%<br>AC 34.5%<br>Other 7.5%	-	-	Preoperative ce-MRI should be performed in patients with large pulmonary lesions (>3 cm diameter or higher than T1 stage) suggestive of NSCLC.

Abbreviations: AC = adenocarcinoma, Asympt. = asymptomatic, BI = brain imaging, BM = brain metastasis/es, ce = contrast enhanced, CT = computed tomography, FU = follow-up, GGO = ground glass opacification, HRCT = high resolution computed tomography, LCC = large cell carcinoma, LCNEC = Large cell neuroendocrine lung carcinoma, molGPA = molecular graded prognostic assessment (molGPA) model, MRI = magnetic resonance tomography, NOS = not otherwise specified, NSCLC = non-small cell carcinoma, PET-CT = Positron-emission computed tomography, SCLC = small cell lung carcinoma, SCC = squamous cell carcinoma, Sympt. = symptomatic, TNM = tumour node metastases, UK = United Kingdom, USA = United States of America.
